# Health behavior patterns of sugar-sweetened beverage consumption among Brazilian adolescents in a nationally representative school-based study

**DOI:** 10.1371/journal.pone.0245203

**Published:** 2021-01-07

**Authors:** Luana Lara Rocha, Milene Cristine Pessoa, Lucia Helena Almeida Gratão, Ariene Silva do Carmo, Cristiane de Freitas Cunha, Tatiana Rezende Prado Rangel de Oliveira, Larissa Loures Mendes

**Affiliations:** 1 Pediatrics Department, Universidade Federal de Minas Gerais, Belo Horizonte, Minas Gerais, Brazil; 2 Nutrition Department, Universidade Federal de Minas Gerais, Belo Horizonte, Minas Gerais, Brazil; 3 Ministry of Health, Brasília, Brazil; 4 Nutrition Department, Pontifícia Universidade Católica de Minas Gerais, Belo Horizonte, Minas Gerais, Brazil; Addis Ababa University, ETHIOPIA

## Abstract

Studies on sugar-sweetened beverage consumption patterns can help in the individual and population level management of chronic non-communicable diseases and other conditions. This study aimed to identify the association between health behavior patterns and the consumption of sugar-sweetened beverages among Brazilian adolescents from a nationally representative school-based study. A cross-sectional study analyzed data from 71,553 adolescents aged 12–17 years who attended public and private schools in Brazilian cities, from the Study of Cardiovascular Risk in Adolescents. Principal component analysis was performed to identify health behavior patterns, and ordered logistic regression was performed to identify the association between health behavior patterns and sugar-sweetened beverage consumption. Sugar-sweetened beverage consumption (mL/day) was used as the dependent variable. The analyses were performed using Stata software version 14.0 with a significance level of 0.05. Patterns 2 (alcoholic beverage and smoking habit) and 3 (ultra-processed food and screen time) of health behaviors and regularly purchasing snacks in the school cafeteria increased the odds of sugar-sweetened beverage consumption, while pattern 1 (water, unprocessed and minimally processed food and physical activity) decreased these odds. The adoption of healthy habits can indirectly stimulate the adoption of other habits beneficial to health. These results indicate the importance of adopting a set of regulatory measures to reduce sugar-sweetened beverage consumption.

## Introduction

Sugar-sweetened beverages (SSBs) refer to any non-alcoholic liquid sweetened with various forms of added sugars, such as sucrose, high-fructose corn syrup, and fruit juice concentrates [1]. According to the NOVA food classification, SSBs are ultra-processed foods because they are composed of industrial formulations, including antioxidants, stabilizers, and preservatives [2]. As a category, SSBs are an important source of calories in adolescents’ diet, particularly in developing countries, due to widespread access, affordability, commercialization, advertising, and subsidies and tax cuts by the governments [3–6].

Studies conducted in other countries have found that adolescents habitually consume SSBs daily [7, 8], and these beverages may represent approximately 18% of total energy [4]. In Brazil, SSBs consumption by adolescents was assessed by the National School Health Survey, which found that approximately 30% of students consumed soft drinks five or more times a week [9].

According to the World Health Organization (WHO), SSBs consumption is a harmful food habit and is considered to be a risk factor for chronic non-communicable diseases (NCD) due to the resulting increase in the intake of free sugars [10]. Excessive consumption of these beverages by adolescents is associated with overweight and obesity [11–15], NCD [16–18], and mental disorders [19–22].

Evaluating adolescents’ behavior patterns and how they influence SSBs consumption is important in identifying axes of action for public policies, regulations, and food and nutrition education, which aims to reduce the availability, purchase, advertising, and consumption of these beverages. This study used data from the Study of Cardiovascular Risk in Adolescents (Portuguese acronym, “ERICA”) and aimed to identify health behavior patterns and their association with the consumption of SSBs among Brazilian adolescents.

## Materials and methods

### Study design

This cross-sectional study analyzed the data from ERICA conducted between March 2013 and December 2014. ERICA is a national cross-sectional school-based study that aimed to estimate the prevalence of cardiovascular risk factors and metabolic syndrome among adolescents aged 12–17 years who attended public and private schools in Brazilian cities with a population of > 100,000 [23]. Detailed information regarding the sampling process, research protocol, and data collection were described in studies by Bloch et al. [24] and Vasconcellos et al. [23].

### Study population and data collection

In this study, 74,589 adolescents from 1,247 schools in 124 Brazilian municipalities were evaluated. The research population was stratified into 32 geographical strata: 26 state capitals, one federal district, and five strata representing other municipalities in each macroregion of the country. The schools were randomly selected according to the number of students enrolled and the distance from the state capital. Three classes were selected per school with different combinations of school schedule times (morning and afternoon) and grades (seventh, eighth, and ninth grades of elementary school; first, second, and third grades of high school). All students in the selected classes were invited to participate. Adolescents who were not aged 12–17 years, or had some degree of disability that hindered them from undergoing the anthropometric assessment and filling out the questionnaire, or were pregnant, were not eligible to participate.

The ERICA sample consisted of 102,327 eligible adolescents, of which 73,160 responded to a 24-h food recall, and 74,589 filled out the self-administered questionnaire using a personal digital assistant (LG GM750Q). The self-administered questionnaire was composed of 105 questions divided into 11 blocks covering the following areas: socioeconomic status, work, smoking status, alcohol consumption, physical activity, medical history, sleeping hours, eating behavior, oral health, common mental disorders, and reproductive health. The variables used in this study were measured using a 24-h food recall and self-administered questionnaire. In total, 71,971 adolescents who had complete data for the adolescent questionnaire and 24-h food recall were evaluated [25].

Food consumption was assessed using a face-to-face 24-h food recall performed by trained interviewers. Brazil Nutri software was used to record food consumption data, with direct recording of information on *netbooks*. The interview technique used was the multiple-pass method [26], which consisted of a five-stage guided interview, aimed at reducing the underreporting of food consumption. The software used contained a list of 1,626 foods and beverages from the 2002 to 2003 household budget survey conducted by the Brazilian Institute of Geography and Statistics (IBGE) [27], and developed by the Ministry of Health in partnership with the Institute of Social Medicine (Federal University of Rio de Janeiro). The database used in the National Dietary Survey was developed by the Brazilian Institute of Geography and Statistics in 2008–2009 [28, 29].

After converting the weight of the food items into grams [28], the dataset was linked to a nutritional composition table [29] to obtain the total daily calorie consumption of each adolescent. The foods were classified based on the degree of processing, as indicated by the NOVA food classification system [2]. This classification system categorizes all foods into four groups based on the nature, extent, and purpose of the industrial processes they undergo: unprocessed and minimally processed foods, processed culinary ingredients, processed foods, and ultra-processed foods [2]. The culinary preparations were dismembered, and the ingredients were classified according to this categorization. The foods were categorized by two independent researchers, and any discrepancies were resolved by an expert researcher.

ERICA was approved by the Research Ethics Committees of the Institute of Studies in Collective Health of the Federal University of Rio de Janeiro (Report 01/2009), in each state of Brazil and the Federal District. All adolescents who agreed to participate provided written informed consent.

### Dependent variable

In this study, SSBs consumption (mL/day) was used as the dependent variable. This variable was calculated from the sum of the quantities of the following types of drinks consumed by adolescents: regular soda, energy drinks, industrialized juices, and chocolate and milk drinks with added sugar and chemical additives (antioxidants, stabilizers, and preservatives). A categorical variable was created from the values of the tertiles of SSBs consumption distribution in mL/day: 1st tertile, 0–7.2 mL/day; 2nd tertile, 8–450 mL/day; and 3rd tertile, 451–3,000 mL/day. Adolescents reporting consumption > 3,000 mL of SSBs daily were excluded from the sample to eliminate outliers that may have been caused by measurement errors; thus, 71,475 adolescents were included in the study. The excluded adolescents did not differ statistically in other study measures from those who were included in the study.

### Independent variables

In the present study, food data, alcohol consumption, physical activity, smoking status, consumption of unprocessed and minimally processed and ultra-processed foods from the recall, and consumption of water were used, and they were all considered as explanatory variables. The independent variables were selected from a literature review to identify possible factors that may be associated with the consumption of sugary beverages.

Regarding information on sociodemographic characteristics, the following were used: gender, race/ethnicity, and age. Socioeconomic score was defined using the Brazil Criterion [30], in which the possession of assets (colored television, radio, bathroom, automobile, refrigerator, freezer, washing machine, and DVD player), presence of a domestic worker, and education of the head of the family [30] were considered. However, in 30.8% of the questionnaires, no information on maternal education was obtained. Thus, we opted for the use of “wealth proxy,” as adopted by Moura [31], renamed as the socioeconomic score, which considered only the possession of assets and the presence of a domestic worker. Thus, rather than using economic class definitions, the socioeconomic score was categorized into three equal intervals (low, 0–12; medium, 13–25; and high, 26–38).

Regarding the food data, the purchase of snacks from the school cafeteria were used. For the “*purchase of snacks from the school cafeteria*” variable, the categories that refer to the “*absence of canteen offer*,” “*do not consume a school canteen snack*,” and “*sometimes consume the school canteen snack*” were grouped in the category “do not consume/irregular consumption,” whereas the categories “*consume canteen snack almost every day*” and “*consume canteen snack every day*” were grouped into “regular consumption”.

The consumption of unprocessed and minimally processed and ultra-processed foods is considered a quantitative variable expressed in grams per day from the 24-h food recall. Regarding ultra-processed foods, SSBs consumption as a dependent variable in this study did not include the ultra-processed food consumption variable. The water consumption variable was obtained by asking “*How many glasses of water do you drink in a day*?” with the answer choices of “*I don't drink water*,” “*1 to 2 glasses per day*,” “*3 to 4 glasses per day*,” and “*5 or more glasses per day*”

The questionnaire used by ERICA to assess the practice of physical activity by adolescents was the Physical Activity Questionnaire for Adolescents (QAFA), validated by Farias Junior et al. [32]. The variable “physical activity practice” was constructed considering the minutes of physical activity performed per week, based on the recommendation of the World Health Organization of 300 min per week [33]. Following the methodology used in the National School Health Survey [9], the physical activity practice variable was categorized into inactive (0 minutes per week), insufficiently active 1: 1–149 minutes per week, insufficiently active 2: 150–299 minutes per week, and active—equal or more than 300 minutes per week.

The screen time variable was obtained using the question “*On a typical weekday*, *how many hours do you use a computer or watch TV or play a video game*?” The answers were categorized as “less than two hours” and “more than two hours” per day in front of screens, according to the recommendations of the American Academy of Pediatrics [34]. Data on consumption of alcoholic beverages were obtained using the question “*In the last 30 days (one month)*, *on how many days did you have at least one glass or one dose of alcoholic beverages*?” The answers were categorized as “not consuming” for those who did not drink any alcoholic beverages or who did not drink on any day of the month before the evaluation, and “consumes” for those who consumed alcoholic drinks at least one day in the previous month. The smoking status variable was obtained using the question, “*Do you currently smoke*?” The answer was either “yes” or “no”.

The variables gender, race/ethnicity, age, socioeconomic score, and school type (public/private) were used to describe the sample and as adjustment variables in the regression model. Moreover, the behavioral variables (screen time, physical activity, smoking status, consumption of alcohol, water, ultra-processed foods and unprocessed and minimally processed foods) were used to build the standards that were be used as explanatory variables.

### Statistical analyses

The descriptive analysis included the calculation of frequencies and measures of position and central tendency. The Shapiro-Wilk test was used to verify the hypothesis of normality of the quantitative study variables.

To identify health behavior patterns that may be associated with adolescents’ consumption of SSBs, principal component analysis (PCA) was performed, which is an exploratory analytical method that condenses the information contained in the original (observed) variables into a smaller number of variables, with minimal loss of information. The Kaiser-Mayer-Olkin (KMO) coefficient was estimated as a measure of adequacy of the PCA, with values between 0.5 and 1.0 considered acceptable for this index. Subsequently, the components with eigenvalues > 1.0, defined according to the scree plot graph, were extracted from the PCA. The structure of the components was obtained from the indicators that presented factor loads > 0.4, with a variable being generated in units of scores for each behavioral pattern. For each pattern, a categorical variable was created from the values of the distribution tertiles of the scores of these patterns.

Bivariate analysis was conducted using an ordered logistic regression model, with the tertile of SSBs consumption as a dependent variable (the lowest tertile of consumption remained as a reference category). The adjustment variables that obtained a p-value < 20% (p<0.20) were used in the ordered logistic regression model. The odds ratio (OR) with 95% confidence interval (95% CI) was used as a measure of effect.

The analyses were performed using Stata software version 14.0 (StataCorp LP, College Station, United States). It is also noteworthy that, in all the performed analyses, the sample complexity was considered using the Stata svy command, with a significance level of 0.05.

## Results

A total of 71,475 adolescents were evaluated: 50.2%, men; 60%, non-white; 35.1%, aged between 12 and 13 years; 74.1%, with average socioeconomic score; 82.8%, from public schools; and 46.7%, purchase snacks in the school cafeteria (Table 1). The prevalence of SSBs consumption was 68% (adolescents who consumed any amount of SSBs on the day evaluated by 24-h food recall).

**Table 1:** Characteristics of Brazilian adolescents evaluated by the ERICA study. Brazil, 2013–2014. (n = 71,475).

Variables	Absolute Frequency	Relative Frequency (%)
*Gender*		
Female	35,587	49.8
Male	35,888	50.2
*Race/Ethnicity*		
White	27,832	40.0
Non-white	41,747	60.0
*Age (years)*		
12–13	25,088	35.1
14–15	25,009	35.0
16–17	21,378	29.9
*Socioeconomic Score*		
High	15,727	23.5
Medium	49,655	74.
Low	1,628	2.4
*School Type*		
Public	59,167	82.8
Private	12,308	17.2
*Food Purchase in the School Cafeteria*		
Non/Irregular	38,068	53.3
Regular	33,407	46.7

The PCA results are shown in Table 2, presenting the three main components, with a contribution of 49.8% of the variance of the total information. The KMO index and factorial loads of all indicators were satisfactory (Table 2). Pattern 1 was characterized by a higher consumption of water (more water glasses) and unprocessed and minimally processed foods and frequent physical activity (more active individuals). Pattern 2 was characterized by more days of alcoholic beverage consumption and a smoking habit. Pattern 3 was characterized by a higher consumption of ultra-processed foods and screen time of more than 2 hours.

**Table 2:** Factor loads of the first three components of the principal component analysis of Brazilian adolescents included in the ERICA study. Brazil, 2013–2014.

Indicators	Pattern 1	Pattern 2	Pattern 3	KMO
Water Consumption	**0.4813**	-0.3708	-0.0399	0.5301
Unprocessed and Minimally Processed Consumption	**0.4655**	-0.1729	0.2348	0.5697
Ultra-processed Consumption	0.1143	0.2771	**0.6327**	0.5523
Screen Hours per Day	-0.0247	0.3724	**0.5480**	0.5469
Physical Activity Practice	**0.6057**	-0.1377	0.0576	0.5259
Alcohol Consumption	0.3156	**0.5832**	-0.2066	0.5045
Smoking	0.2673	**0.5080**	-0.4434	0.5088
*Eigenvalue*	1.24	1.22	1.03	-
*Explained variance (%)*	17.6	17.4	14.8	-
*Cumulative variance explained (%)*	17.6	35.0	49.8	-
*Overall*	-	-	-	0.5248

KMO: Kaiser-Meyer-Olkin

The variables associated with SSBs consumption in the ordered logistic regression model are shown in Table 3. Regularly purchasing snacks in the school cafeteria (OR, 1.19; 95% CI, 1.05–1.35), and being classified as either the second (OR, 1.24; 95% CI, 1.10–1.40) or third (OR, 1.70; 95% CI, 1.47–1.97) tertile of pattern 2 of health behaviors, and being classified as either the second (OR, 1.20; 95% CI, 1.07–1.33) or third (OR, 1.24; 95% CI, 1.09–1.41) tertiles of pattern 3 of health behaviors increased the odds of SSBs consumption in teenagers who consumed between 8 mL and 450 mL of SSBs per day (second tertile) (Table 3). However, being the third (OR, 0.89; 95% CI, 0.79.0.99) tertile of pattern 1 of health behaviors decreased the odds of SSBs consumption in adolescents who were in the second tertile of SSBs consumption (Table 3).

**Table 3:** Simple and multiple ordered logistic regression analyses: Odds of sugar sweetened beverage consumption by Brazilian adolescents included in the ERICA study. Brazil, 2013–2014.

Variables	Sugar Sweetened Beverages Consumption Tertile			
	OR^1^ (CI 95%)^a^		OR^2^ (CI 95%)^a^^,^^b^	
	2^nd^ tertile (8–450 mL)	3^rd^ tertile (451–3.000 mL)	2^nd^ tertile (8–450 mL)	3^rd^ tertile (451–3.000 mL)
*Food Purchase in the School Cafeteria*				
Non/Irregular	1.0	1.0	1.0	1.0
Regular	1.26 (1.12–1.41)***	1.12 (1.01–1.24)*	1.19 (1.05–1.35)**	1.13 (1.02–1.26)*
*Pattern 1*				
Tertile 1	1.0	1.0	1.0	1.0
Tertile 2	0.91 (0.82–1.01)	0.98 (0.89–1.08)	1.02 (0.91–1.13)	1.04 (0.93–1.17)
Tertile 3	0.83 (0.76–0.91)***	0.91 (0.82**–**1.01)	0.89 (0.79–0.99)*	0.87 (0.77–0.99)*
*Pattern 2*				
Tertile 1	1.0	1.0	1.0	1.0
Tertile 2	1.39 (1.27–1.53)***	1.41 (1.26–1.57)***	1.24 (1.10–1.40)***	1.32 (1.17–1.48)***
Tertile 3	1.87 (1.67–2.09)***	1.93 (1.73–2.15)***	1.70 (1.47–1.97)***	1.86 (1.63–2.11)***
*Pattern 3*				
Tertile 1	1.0	1.0	1.0	1.0
Tertile 2	1.32 (1.19–1.47)***	1.27 (1.12–1.44)***	1.20 (1.07–1.33)**	1.14 (1.01–1.28)*
Tertile 3	1.43 (1.29–1.59)***	1.50 (1.35–1.65)***	1.24 (1.09–1.41)**	1.24 (1.11–1.40)***

OR: Odds Ratio; CI: Confidence Interval; mL: milliliters

^1^Simple ordered logistic regression model

^2^Multiple ordered logistic regression model

^a^1^st^ tertile (0–7.2 mL) as a reference

^b^Adjusted for the variables gender, race/ethnicity, age, socioeconomic score, and school type

*p<0.05

**p<0.01

***p<0.001

Regularly purchasing snacks in the school cafeteria (OR, 1.13; 95% CI, 1.02–1.26), and being classified as either the second (OR, 1.32; 95% CI, 1.17.1.48) or third (OR, 1.86; 95% CI, 1.63–2.11) tertiles of pattern 2 of health behaviors, and being classified as either the second (OR, 1.14; 95% CI, 1.01–1.28) or third (OR, 1.24; 95% CI, 1.11–1.40) tertiles of pattern 3 of health behaviors increased the odds of SSBs consumption in teenagers who consumed between 451 mL and 3,000 mL of SSBs per day (third tertile) (Table 3). However, being classified as the third tertile (OR, 0.87; 95% CI, 0.77–0.99) of pattern 1 of health behaviors decreased the odds of SSBs consumption in adolescents who were in the third tertile of SSBs consumption (Table 3).

## Discussion

In this cross-sectional study on Brazilian adolescents, we evaluated the association between SSBs consumption and variables of health behavior patterns. The pattern 1 was characterized by a higher consumption of water and unprocessed and minimally processed foods and frequent physical activity. Pattern 2 was characterized by more days of alcoholic beverage consumption and a smoking habit. Pattern 3 was characterized by a higher consumption of ultra-processed foods and screen time of more than 2 hours. More than half of the adolescents included in the study consumed SSBs, and patterns 2 and 3 of health behaviors and regularly purchasing snacks in the school cafeteria increased the odds of SSBs consumption, whereas pattern 1 decreased the odds of SSBs consumption.

The odds of SSBs consumption were higher in adolescents who regularly purchased food from the school cafeteria. Generally, ultra-processed foods, such as SSBs, savory snacks, and biscuits, are available in school cafeterias, as reported in other studies [2, 35, 36], increasing the consumption of these foods [35–37]. The availability of healthy food in the school environment is associated with less consumption of unhealthy foods [36], highlighting one of the ways to decrease unhealthy food consumption.

The WHO in 2016 published a document with a series of recommendations for reducing sugary drink consumption among children and adolescents in schools [38]. The recommendations of this document for the school food environment are also applicable to the reduction of SSBs consumption. The recommendations are to increase the availability of safe water in schools, carry out food and nutritional education actions to make children and adolescents aware of healthy beverage options, reduce the availability of SSBs in the school environment, and ban the marketing of SSBs in schools [47].

Regarding health behavior patterns, belonging to pattern 1, characterized by a higher consumption of water and unprocessed and minimally processed foods and frequent physical activity, decreased the odds of SSBs consumption. In the literature, studies that analyze SSBs consumption and healthy behavior patterns in adolescents are limited, and generally these studies analyze these behaviors in isolation. For instance, when water consumption was assessed separately, it was found to be associated with lower SSBs consumption, as in our study [39]. Regarding unprocessed and minimally processed food consumption, there are no studies in the literature that evaluate their association with the consumption of SSBs. Thus, this is the first study to trace the relationship between the consumption of unprocessed and minimally processed foods and consumption of SSBs. In a pattern that also considers water consumption and physical activity practices, increased consumption of unprocessed and minimally processed foods can reduce the consumption of SSBs.

However, when physical activity was assessed separately, it was generally found that physical activity is a factor that increases SSBs consumption [40–44], a result that can be attributed to the association between the consumption of sports and energy drinks and physical activity among adolescents [43, 45, 46]. Meanwhile, when increased physical activity is combined with a healthy diet, it can help reduce the consumption of SSBs, as shown in this study.

This study showed that the adoption of healthy eating habits reduced SSBs consumption in adolescents and modified the effect of physical activity on the same. The joint analysis of variables through behavioral patterns is relevant because the grouping of factors in an individual can have a greater effect than the sum of the effects analyzed in isolation [47, 48].

Pattern 2 of health behaviors was characterized by more days of alcoholic beverage consumption and a smoking habit. Other studies found similar results when analyzing these factors separately [49–51]. SSBs consumption is accompanied by several health risks, in which alcohol consumption and smoking are considered as not only as gateways for the use of other substances [19], but also risk factors for the development of mental disorders [19–22]. Thus, measures that reduce SSBs consumption as well as the use of alcohol and tobacco, are essential in ensuring adolescents’ physical and psychological health.

Pattern 3 of health behaviors was characterized by a higher consumption of ultra-processed foods and screen time of more than 2 hours. When analyzing these factors separately, similar results were found for ultra-processed food consumption and screen time [43, 50, 52–55]. The association between ultra-processed foods and SSBs consumption is particularly strong because these drinks are included in the ultra-processed food category [2, 56]. Therefore, it is expected that higher consumption of these foods will be associated with higher SSBs consumption.

Screen time, which is included in pattern “3” of health behaviors, increases the likelihood of SSBs consumption. This may be explained by the exposure of adolescents to advertisements for ultra-processed foods and beverages in the media and increased sedentary behavior [57, 58]. Therefore, this set of risk factors can influence the consumption of these foods by adolescents [57, 58]. Guimarães et al. [59] analyzed the extent and nature of food and beverage advertising on the three main free television channels in Brazil, and found that 9 of 10 food and beverage advertisements presented ultra-processed foods, with soft drinks the most heavily represented product. Thus, these results show the importance of public policies that regulate the advertising of foods and ultra-processed beverages to children and adolescents [57, 58].

To reduce SSBs consumption among adolescents and their effects on the health of the individual, it is necessary to adopt a set of regulatory measures and educational actions. Gortmaker et al. [60] developed an evidence review process and microsimulation model to assess the profitability of interventions for childhood obesity and found that SSBs taxation, elimination of subsidies, reduction in advertising of foods considered unhealthy, and regulation of the sale of food in schools (setting nutritional standards for foods and drinks) were the most cost-effective measures for reducing childhood obesity. As evidenced in this study, and in that of Azeredo et al. [36], the adoption of a healthy diet as well as greater availability of healthy food in the environment, and reduced availability of ultra-processed food and beverages, can also be effective measures to reduce SSBs consumption.

These measures have already been applied in other countries and have been shown to be effective in reducing SSBs consumption [36, 39, 61–65]. Their application in Brazil has an urgent need, in view of the increasing rates of overweight, obesity, and NCD among adolescents.

This study has some limitations. For example, the 24-h food recall may not accurately characterize actual habitual consumption among adolescents and can lead to bias in the dietary assessment, including misreporting. However, the representative sample (71,533 adolescents) allows for a better generalization to the larger Brazilian population of adolescents aged between 12 and 17 years and residing in cities with > 100,000 inhabitants. In addition, this was the first study to evaluate the association between health behaviors on aggregate and SSBs consumption.

## Conclusions

Overall, the data showed that adolescents who are in 2^nd^ and 3^rd^ health behavior patterns (related to unhealthy practices) and buy snacks regularly in school cafeterias have greater odds of SSBs consumption. However, those characterized in the 1^st^ behavioral pattern (related to healthy practices) had reduced odds of consumption. Thus, the adoption of healthy habits can indirectly stimulate the adoption of other habits beneficial to health.

These results indicate the importance of adopting a set of regulatory measures to reduce SSBs consumption, such as the taxation of these drinks, prohibition of sales in and around schools, and increasing the availability of healthy beverages and foods in the school environment. The adoption of interventions in isolation, to address each risk factor separately, may not have the same effect as broad interventions that consider the complexity of the problem.
